# Mirfin Mpundu: accessing medicines, fighting resistance

**DOI:** 10.2471/BLT.19.030419

**Published:** 2019-04-01

## Abstract

Mirfin Mpundu spoke to Gary Humphreys about his experiences in African countries and in the United States of America, the prospects for containing antimicrobial resistance, and the contribution that faith-based organizations make to health systems in African countries.

Q: How did you first become interested in the field of medicines access and quality?

A: I didn’t just become a pharmacist because I thought it would be interesting. I really wanted to make a difference in people’s lives and my early experiences working in Zambia only confirmed that desire. 

Q: Can you talk about those experiences?

A: I worked as a pharmacist for the Churches Medical Association of Zambia in Lusaka for two years. My activities included working on procurement and distribution of essential medicines and medical supplies to health facilities and I also participated in developing a central distribution warehouse for mission hospitals. I learnt a lot about forecasting and supply-chain planning, especially regarding HIV and malaria commodities. I also worked on logistics development to assure supplies to mission hospitals. On the stewardship side, I was involved in some educational projects related to HIV that were being run by donor agencies and the health ministry, and I conducted training in rational use of drugs for nursing students. 

Q: You left for the United States of America (USA) in 2000. What did you do there?

A: I worked as a pharmacist in the hospital systems in the USA in different roles. For example, I was the Director of Critical Care Services and the Emergency Department at Laurel Regional Hospital in Maryland. It was interesting work and quite fulfilling, but I knew I had to get back to my public health roots at some point.

Q: Can you say more about that?

A: Well, that was a big reason for getting into the pharmaceuticals field in the first place, to work among the most needy in hard-to-reach areas. Even though I was working in the USA, I knew that the issues that I had left behind in Zambia were still there. Stock-outs, lack of trained staff, poor forecasting and quantification, wasted resources, lack of evidence-based practices both in clinical practice and in the supply chains – as in other African countries. I knew that I could do something about it and thought that I could leverage the experience I had gained in the West.

Q: So, you went back home?

A: Yes, in 2013 – a year later – I joined the Ecumenical Pharmaceutical Network (EPN).

“A significant portion of health services are provided by the churches in Africa.”

Q: Can you explain what that is?

A: It’s a network of faith-based organizations with members in 37 countries. Our main mandate is to support members in promoting access to quality-assured medicines and pharmaceutical services. The EPN also hosts Action on Antibiotic Resistance (ReAct), Africa, a civil society organization focused on antibiotic resistance. 

Q: How do the two organizations work together?

A: The EPN is working on supporting the establishment of antimicrobial stewardship programmes, and infection prevention and control programmes in faith-based health facilities, as well as ensuring availability of quality-assured antibiotics. At the policy level, EPN and ReAct advocate for sustainable solutions in addressing antimicrobial resistance based on WHO’s *Global action plan on antimicrobial resistance*. ReAct also works alongside African countries, offering technical assistance as they develop their national action plans. I personally have been very active in this area, supporting the development of national action plans in Zimbabwe, but also in Ghana, Kenya, Malawi, Nigeria and Zambia. In collaboration with colleagues, I also work on awareness raising and knowledge sharing using the ReAct annual conference platform. That platform brings together champions on antimicrobial resistance from different African countries to discuss and share lessons. My work with EPN is focused on advocacy, noncommunicable diseases, maternal and child health, and HIV. Part of that work involves health workforce capacity building. I also work on a lot of my own projects, including antimicrobial stewardship and infection prevention and control programmes in the Democratic Republic of the Congo (DRC). The DRC programmes include facilities that treat people with Ebola. I was there a couple of months before the outbreak, working on infection prevention and control, and antimicrobial stewardship. I am not a desk person. I like to get my ‘hands dirty’, as they say. I am also leading a project in Zimbabwe that is focused on care for people with diabetes that is delivered through a network of churches.

Q: What is the contribution of faith-based organizations in delivering health services?

A: A significant portion of health services are provided by the churches in Africa, with some studies putting the contribution as high as 60%. 

Q: Can you explain what churches do?

A: It varies from church to church and, of course, I’m talking about the various ancillary institutions to churches, such as schools and hospitals. Most church-run health facilities are in the rural and hard-to-reach areas and they provide various services; emergency, surgery, internal medicine, urology, pharmacy, maternal, neonatal and child health services and others. This contribution is not always recognized.

Q: Why do you think that is?

A: I think partly because a lot of the work the churches are doing takes place in remote under-resourced places where governments struggle to provide services. So the churches’ activity tends to be out of the spotlight, so to speak. Also, actors in the faith-based health sector tend not to publish what they are doing. As a result, the public is unaware of how much they affect people's lives. 

Q: Are donors aware of the work you are doing?

A: Traditionally, donors have tended to only support government health facilities, but that is changing now as we see faith-based health systems being recipients of grants such as for HIV, vaccination and malaria.

Q: When did you first become aware that antimicrobial resistance was a problem?

A: I think I first began to really notice when I was working in Maryland. I could see treatment failures, and it was quite clear to me that there was an issue. It made me reflect on some earlier cases I'd had in Zambia, which involved untreatable sepsis. It also made me think about my own mother’s death. She acquired an infection in the hospital after delivering my sister by C-section. She was a healthy woman and she walked into a hospital and never came back. Did the antibiotics fail? That’s a question I have asked myself many times. Now, antimicrobial resistance is something we see in all the big diseases.

“We now have about 25 countries in Africa that have national action plans, but there are only three or four that are actually implementing them.”

Q: Can you say more about that?

A: With regard to HIV, for example, levels of resistance to certain antiretrovirals have continued to increase in all regions, reaching and even exceeding one in 10 cases in several countries. Some countries have reported levels of drug resistance at or above 15% among people starting HIV treatment, and levels as high as 40% among people re-starting treatment. Drug-resistant tuberculosis is also a major concern, both in terms of multidrug-resistant tuberculosis (MDR-TB) and extensively drug-resistant TB (XDR-TB).

Q: Can you explain the difference between the two?

A: MDR–TB is a form of tuberculosis caused by bacteria that are resistant to the two most powerful, first-line anti-TB drugs (isoniazid and rifampicin) and has to be treated using second-line drugs that are expensive and toxic. XDR–TB is a more serious form of MDR–TB. The bacteria that cause it are resistant to even the most effective second-line drugs. The most recent estimate is that, worldwide in 2017, around a half million people developed tuberculosis that was resistant to rifampicin, which is the most effective first-line drug. Of that group, roughly 80% had MDR–TB. India, China and the Russian Federation accounted for almost half of the world’s cases, but there are epidemics of drug-resistant tuberculosis on the African continent also, notably in West African countries and in South Africa. 

Q: What about malaria?

A: The research shows that artemisinin-based combination therapies remain effective in 95% of cases, except in the Greater Mekong Sub-region. However, resistance to the four commonly used insecticides used for mosquito control – I’m talking about the pyrethroids, organochlorines, carbamates and organophosphates – is widespread and has been observed in all major malaria vectors across the WHO regions of Africa, the Americas, South-East Asia, the Eastern Mediterranean and the Western Pacific.

Q: How optimistic are you that current plans and initiatives will be sufficient to contain antimicrobial resistance?

A: I am optimistic by nature and am committed to realizing the ReAct vision – a world free from fear of untreatable infections. However, I can only be cautiously optimistic about our ability to make progress regarding antimicrobial resistance. One of the reasons is the lack of action or limited action on the ground in many countries. We now have about 25 countries in Africa that have national action plans, but there are only three or four that are actually implementing them. Similarly, we still have a long way to go in terms of mainstreaming antimicrobial resistance initiatives into health systems. This is notably true in regard to work on malaria, HIV and tuberculosis. All these programmes continue to run as silos, rarely sharing resources, whether these represent information, supply chains, laboratory facilities or surveillance. 

Q: What can be done to change this situation?

A: Hopefully the Interagency Coordination Group on Antimicrobial Resistance recommendations due this September will give the impetus we need to keep the flame burning.

